# Electronic Structures and Photodetachment of TeO_2_^−^, TeO_3_^−^, and HTeO_4_^−^ Anions: A Cryogenic Photoelectron Spectroscopic Study

**DOI:** 10.3390/molecules30183757

**Published:** 2025-09-16

**Authors:** Fan Yang, Xueying Li, Peng Tang, Qixu Zhao, Jian Zhang, Ye Mei, Zhubin Hu, Zhenrong Sun, Yan Yang

**Affiliations:** 1 State Key Laboratory of Precision Spectroscopy, School of Physics and Electronic Science, East China Normal University, Shanghai 200241, Chinaymei@phy.ecnu.edu.cn (Y.M.); zbhu@lps.ecnu.edu.cn (Z.H.); 2 College of Chemistry & Chemical Engineering, Donghua University, Shanghai 201620, China; zhangjian@dhu.edu.cn; 3 Collaborative Innovation Center of Extreme Optics, Shanxi University, Taiyuan 030006, China

**Keywords:** anion photoelectron spectroscopy, photodetachment, telluride oxides, density functional theory, photodissociation

## Abstract

Cryogenic anion photoelectron spectroscopy combined with quantum chemical calculations was employed to investigate the electronic structures and photodetachment properties of TeO_2_^−^, TeO_3_^−^, and HTeO_4_^−^ anions. The adiabatic/vertical detachment energies (ADEs/VDEs) of these anions were determined through the photoelectron spectra at 193 nm, yielding values of 2.13/1.94, 4.20/3.64, and 5.64/5.20 eV, respectively. These results align well with the theoretical calculations and were further validated through Franck–Condon factor (FCF) simulations. TeO_2_^−^ and TeO_3_^−^ exhibit a notable multi-reference character, with TeO_3_^−^ showing a pronounced structural change upon detachment from C_3v_ to D_3h_ geometry, leading to a substantial difference between its ADE and VDE. Orbital analyses of the photodetachment processes reveal a progressive shift in the primary contribution to the detached electron—from the Te atom to the O atoms—as the anion size increases. Moreover, a two-photon photodissociation–photodetachment process was identified for HTeO_4_^−^. These findings provide fundamental insights into the geometric and electronic structures of gas-phase tellurium oxides, offering a benchmark for further theoretical modeling and material development involving chalcogen oxide anions.

## 1. Introduction

As representative systems among main-group p-block element oxides, chalcogen oxides exhibit unique electronic configurations and coordination characteristics, making them ideal model compounds for investigating structure–property relationships [1,2,3,4,5]. In particular, tellurium oxides and their derivatives—tellurates—have demonstrated significant potential in acousto-optic devices [6,7,8,9], low-temperature detectors [10], nonlinear optics [11,12,13,14,15,16], and industrial catalysis [17,18,19]. In the semiconductor sector, tellurium oxides are also considered promising candidates for the development of novel amorphous p-type semiconductors [20].

Despite these wide-ranging applications, experimental data on the electron affinities (EAs) and electronic structures of tellurium oxides remain scarce, mainly due to the difficulty in generating gas-phase tellurium oxide anions efficiently. To date, only limited experimental studies have been reported. For example, TeO_2_ has been experimentally shown to have an electron affinity (EA) > 2.2 eV [21], while theoretical calculations predict values between 2.17 and 2.20 eV [22]. This discrepancy highlights the need for more accurate experimental characterization. Furthermore, a systematic understanding of the detachment energies and orbital characteristics of tellurium oxyanions is essential, as these quantities directly affect the photoelectric behavior, catalytic activity, and optical properties of tellurium oxide materials.

In this study, we systematically investigated the TeO_2_^−^, TeO_3_^−^, and HTeO_4_^−^ anions using cryogenic negative-ion photoelectron (NIPE) spectroscopy in combination with quantum chemical calculations. The fragmental TeO_2_^−^ and TeO_3_^−^ anions were generated via collision-induced dissociation (CID) of HTeO_4_^−^ anions within a two-stage ion funnel (Figure S1). The HTeO_4_^−^ were produced by electrospray ionization (ESI) from a 5 mM aqueous solution of H_6_TeO_6_ prepared in a H_2_O:CH_3_CN = 1:2 solvent mixture. The corresponding detailed spectral features, optimized geometries, and photodetachment orbital characteristics of these species are reported. We provide comprehensive analyses of their adiabatic and vertical detachment energies (ADEs/VDEs) and demonstrate how orbital contributions shift with increasing anion size. These benchmark results not only address existing uncertainties but also contribute to the broader understanding necessary for the development of tellurium oxides in technology-driven applications.

## 2. Results and Discussion

### 2.1. Photoelectron Spectra of TeO_2_^−^, TeO_3_^−^, and HTeO_4_^−^

**Figure 1:**
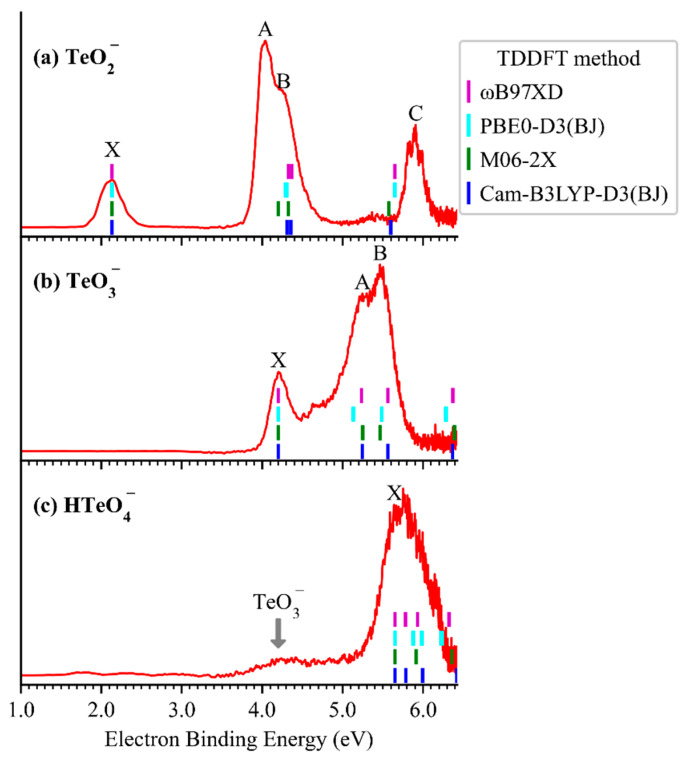
The 13K NIPE spectra (red traces) of TeO_2_^−^ (**a**), TeO_3_^−^ (**b**), and HTeO_4_^−^ (**c**) at 193 nm. Short vertical bars denote the calculated excited-state energies of the neutrals based on various TDDFT methods with the aug-cc-pVTZ(-PP) basis set.

Figure 1 presents the cryogenic negative-ion photoelectron spectra (red traces) of TeO_2_^−^, TeO_3_^−^, and HTeO_4_^−^ at 13 K using a 193 nm (6.424 eV) laser. Each spectrum exhibits a distinct set of peaks, labeled X, followed by A to C, in order of increasing electron binding energy (eBE). These features correspond to photodetachment transitions from the anionic ground state to the ground and excited states of the corresponding neutral species. The vertical detachment energies (VDEs), determined from the maxima of each X peak, are 2.13 eV (TeO_2_^−^), 4.20 eV (TeO_3_^−^), and 5.64 eV (HTeO_4_^−^), respectively. Due to the absence of well-resolved vibrational structures in the X bands, the adiabatic detachment energies (ADEs) were estimated by adding the instrumental resolution to the onset eBE of each X peak. The resulting ADEs are 1.94 eV for TeO_2_^−^, <4.01 eV for TeO_3_^−^, and 5.20 eV for HTeO_4_^−^, respectively. It should be noted that this method is only reliable when minimal structural changes occur upon photodetachment. In cases where substantial structural reorganization occurs—such as for TeO_3_^−^—the ADE may be inaccurately determined or undefined, as will be discussed in more detail in later sections. Additionally, the spectrum of HTeO_4_^−^ shows a distinct band at 4.2 eV, which is attributed to a two-photon photodissociation–photodetachment process. This feature and its mechanistic interpretation will also be elaborated upon in subsequent discussions.

### 2.2. Optimized Structures and Calculated ADEs and VDEs

**Figure 2:**
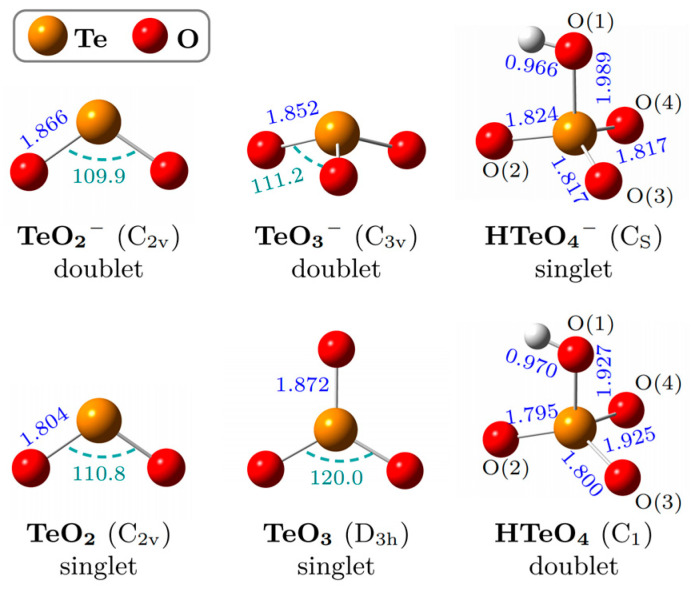
The lowest-energy geometries of TeO_2_^−^, TeO_3_^−^, and HTeO_4_^−^ and the corresponding neutrals. Selected bond lengths (blue, in Å) and bond angles (green, in deg) are provided.

Figure 2 illustrates the lowest-energy structures of TeO_2_^−^, TeO_3_^−^, and HTeO_4_^−^, along with their corresponding neutral species, optimized at the B3LYP-D3(BJ)/aug-cc-pVTZ-(pp) level of theory (see Table S1 for coordinates). TeO_2_^−^ adopts a C_2v_-symmetric V-shaped structure (Te–O = 1.866 Å; ∠O–Te–O = 109.90°) with a doublet ground state. Its neutral counterpart retains the same symmetry, with slightly shortened Te–O bonds (1.804 Å) and a marginally widened bond angle (110.8°), consistent with prior reports [22]. TeO_3_^−^ exhibits a C_3v_-symmetric triangular pyramid (Te–O = 1.852 Å; ∠O–Te–O = 111.2°) with doublet multiplicity. Its neutral form is a D_3h_-symmetric planar triangle (Te–O = 1.872 Å, ∠O–Te–O = 120.0°). The marked structural transformation upon photodetachment, similar to that seen in SO_3_^−^ systems [2,3,23], hinders accurate experimental determination of the ADE for TeO_3_^−^ and necessitates Franck–Condon factor (FCF) analysis. Both HTeO_4_^−^ and HTeO_4_ exhibit approximately tetrahedral geometries. The anion features C_s_ symmetry, with atoms H–O(1)–Te–O(2) forming the mirror plane. The calculated bond lengths of Te–O(1)/O(2)/O(3)/O(4) are 1.989/1.824/1.817/1.817 Å, whereas the neutral species exhibits slightly shorter bonds of 1.927/1.795/1.800/1.925 Å. Notably, the structure of HTeO_4_^−^ undergoes minor distortion upon photodetachment, resulting in loss of symmetry in the neutral HTeO_4_. This behavior contrasts with that of HSO_4_^−^, as the symmetry of HSO_4_^−^ and its neutral counterpart remains essentially unchanged after photodetachment [5]. In addition, the hydrogen in HSO_4_^−^ lies in the opposite position of the O in the symmetry plane, forming a Z-shaped symmetry plane [5], unlike the C-shaped mirror configuration observed in HTeO_4_^−^.

Theoretical VDEs and ADEs calculated for all species are compared with experimental values in Table 1. For TeO_2_^−^ and TeO_3_^−^, commonly used density functional theory (DFT) methods—B3LYP-D3(BJ), PBE0-D3(BJ), M06-2X, ωB97XD, and TPSSh-D3(BJ)—exhibit unsatisfactory agreement with experimental results. These discrepancies are likely due to the multi-reference character of these open-shell systems, as indicated by T1 diagnostic values of 0.026 for TeO_2_^−^ and 0.028 for TeO_3_^−^, both exceeding the 0.02 threshold (see Table S2). To better describe such systems, we employed the MN15L and r2SCAN-3c functionals, which are more suitable for systems with significant multi-reference effects. These methods effectively improved the agreement with the experiment, reducing deviations in ADEs and VDEs to within 0.12 eV. In the case of HTeO_4_^−^, which lies near the diagnostic threshold (T1 = 0.200), acceptable performance was achieved only using the ωB97XD functional. For a more balanced and reliable description for VDE, we also applied the IP-EOM-CCSD method, which yielded a mean absolute error of 0.17 eV and demonstrated good overall consistency with experimental observations. It is worth noting that the discrepancy between theoretical ADE and VDE for TeO_3_^−^ exceeds 0.5 eV, attributable to the substantial geometric change from pyramidal (C_3v_) anion to the planar (D_3h_) neutral, which complicates the accurate estimation of the ADE.

### 2.3. Molecular Orbital and Photodetachment Analyses

To gain deeper insights into the photodetachment mechanisms of the TeO_2_^−^, TeO_3_^−^, and HTeO_4_^−^ anions, we performed natural population analysis (NPA) to examine their charge distributions. As summarized in Table 2, the Te atoms in all three anions exhibit relatively positive charges, which increase progressively with the number of oxygen atoms. In contrast, the O atoms show comparably negative charges, being −0.50 for TeO_2_^−^, −1.14 for TeO_3_^−^, and approximately −1.1 for HTeO_4_^−^. Upon photodetachment, the charge on the Te atom changes by +0.76, +0.58, and −0.05 for TeO_2_^−^, TeO_3_^−^, and HTeO_4_^−^, respectively. Correspondingly, the charges on O atoms increase by +0.24, +0.42, and +1.03, suggesting a progressive shift in the origin of the detached electrons from the Te center to the O atoms as the degree of oxidation increases. This redistribution is further corroborated by molecular orbital (MO) analyses (Figure S2). The oxygen contributions to the highest occupied molecular orbitals (HOMOs) increase from 34.2% in TeO_2_^−^ to 62.7% in TeO_3_^−^ and 97.8% in HTeO_4_^−^, indicating that the electrons involved in photodetachment are increasingly localized on oxygen atoms as the ionic species become more oxygen-rich.

**Figure 3:**
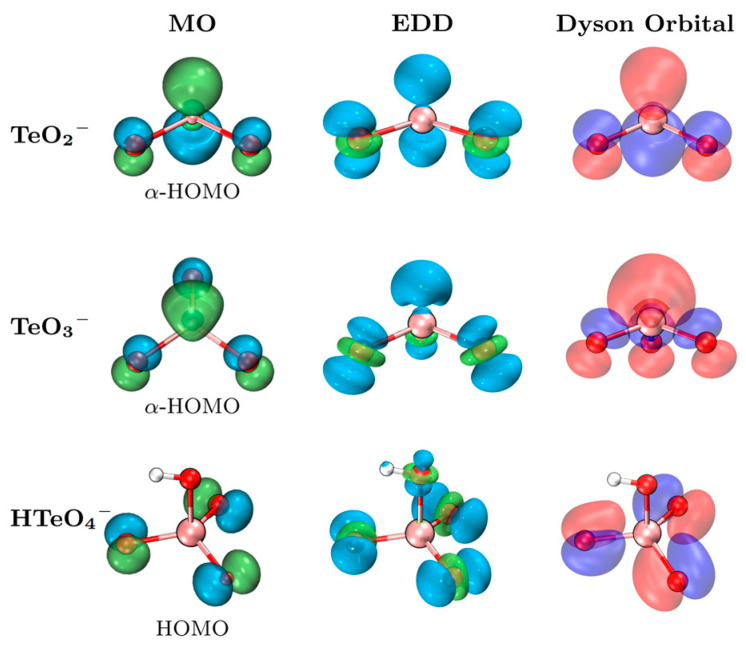
MOs (isovalue 0.08), EDDs (isovalue 0.005), and Dyson orbitals (isovalue 0.15) of the TeO_2_^−^, TeO_3_^−^, and HTeO_4_^−^ anions. Green (blue) represents the increase (decrease) in EDD.

To better elucidate the nature of the photodetachment orbitals, electron density difference (EDD) maps and Dyson orbital analyses were performed and compared with the HOMOs, as shown in Figure 3. The EDD and Dyson orbitals offer reliable characterizations of the electronic emission orbitals from the perspectives of density variation and transition amplitude, respectively [24]. The regions of electron density depletion (blue) in the EDD maps exhibit strong spatial overlap with the corresponding HOMOs, confirming the applicability of the Koopmans’ framework in describing the photodetachment process. Furthermore, the close resemblance between the Dyson orbitals and HOMOs further substantiates that MO analyses provide a reliable qualitative representation of the photodetachment characteristics.

To interpret the high-eBE spectral features, we conducted time-dependent density functional theory (TDDFT) calculations using CAM-B3LYP, PBE0-D3(BJ), M06-2X, and ωB97XD functionals with the aug-cc-pVTZ(-pp) basis set, based on the optimized anion geometries. As shown in the stick spectra of Figure 1, the calculated excited-state transitions align well with the experimental X peaks and show consistent energy shifts corresponding to vertical excitation energies. The TDDFT simulated spectra reproduce most of the observed spectral features (see Table S3 for details), supporting the reliability of the orbital assignments.

### 2.4. Vibrational Excitation and FCF Simulation

**Figure 4:**
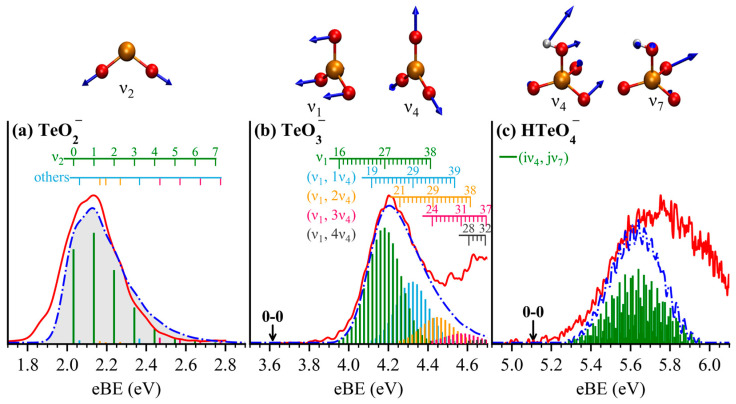
FCF simulated spectra (blue dot-line traces) and the corresponding vibration modes overlaid with the experimental photoelectron spectra (red traces) of TeO_2_^−^ (**a**), TeO_3_^−^ (**b**), and HTeO_4_^−^ (**c**). Gaussian broadenings of 120, 40, and 13 meV were applied to the stick spectra (colored vertical sticks) of TeO_2_^−^, TeO_3_^−^, and HTeO_4_^−^, respectively, in accordance with the instrument resolutions at different electron kinetic energies adopted in the experiments. Detailed vibrational assignments for each system are provided in Tables S4–S6.

To interpret the vibrational structures observed in the experimental spectra and to extract more accurate ADEs, Franck–Condon factor (FCF) simulations were carried out for transitions from the anionic ground states to the corresponding neutral ground states. These simulations provide detailed insights into vibrational fine structures and intensity distributions, wherein the 0–0 transition directly defines the ADE (equivalent to the electron affinity of the neutral species). As shown in Figure 4, the simulated stick spectra were grouped by dominant vibrational mode contributions and convoluted with Gaussian functions using full width at half maximum (FWHM) of 120 meV, 40 meV, and 13 meV for TeO_2_^−^, TeO_3_^−^, and HTeO_4_^−^, respectively, consistent with the instrument resolution at different electron kinetic energies. In each panel, the red solid trace represents the experimental spectrum, the blue dot-dash trace corresponds to the broadened simulated spectrum, and the colored sticks indicate contributions from individual vibrational transitions. Detailed vibrational assignments are summarized in Tables S4–S6, and the vibrational modes involved are visualized in Figure S3.

For TeO_2_^−^ (Figure 4a), the FCFs reveal that the vibrational progression is dominated by the symmetric stretching mode $\nu_{2}$ with a frequency of 826 cm^−1^ (Table S4), while contributions from other modes are negligible. The ADE derived from the 0–0 transition is 2.03 eV, in agreement with both experimental values and previous theoretical predictions [22].

For TeO_3_^−^ (Figure 4b), the spectrum is mainly governed by the umbrella-like $\nu_{1}$ vibration mode (169 cm^−1^) and its coupling with the planar symmetric Te–O stretching mode $\nu_{4}$ (813 cm^−1^). The simulated peak maximum agrees well with the experimental VDE. Vibrational levels ν = 0–15 of the $\nu_{1}$ mode exhibit negligible FCF intensity and thus explain the absence of well-defined features in this region. In contrast, levels from ν = 16 to 38 contribute significantly to the FCFs (Table S5). The overall envelope results from overlap between progressions involving $\nu_{1}$ mode alone, and $\nu_{1}$ coupled $\nu_{4}$ effectively, successfully reproducing the structural features of the experimental X peak. The ADE determined from the 0–0 transition is 3.62 eV, aligning well with predictions from MN15L and r2SCAN-3c functionals in Table 1. Notably, the vibrational behavior of TeO_3_^−^ closely resembles that of SO_3_^−^, due to their analogous geometries and normal mode patterns [2].

In the case of HTeO_4_^−^ (Figure 4c), the X band arises from a complex coupling between the $\nu_{4}$ (227 cm^−1^) and $\nu_{7}$ (540 cm^−1^) modes, which involve both Te–O and H–O intricate stretching and bending vibrations. The simulated spectrum captures the vibrational fine structure near the rising edge of the X band, wherein the primary vibrational interval is mainly arising from the $\nu_{4}$ mode. The ADE extracted from the 0–0 transition is 5.11 eV, in excellent agreement with the calculated value using the ωB97XD functional, as shown in Table 1.

### 2.5. Two-Photon Photodissociation–Photodetachment of HTeO_4_^−^

**Figure 5:**
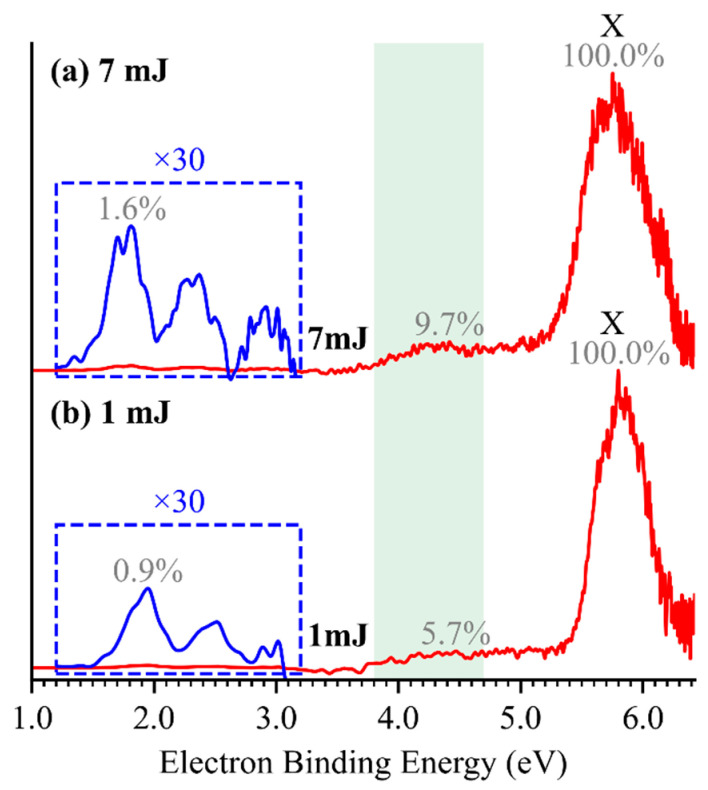
The photoelectron spectra of HTeO_4_^−^ at 193 nm with pulse energies of 1 mJ (**a**) and 7 mJ (**b**).

A series of weak photoelectron signals were observed in the low-eBE region of 3.5–5.0 eV for HTeO_4_^−^ (Figure 1c). These features are hypothesized to arise from photodetachment of fragment anions generated via photodissociation of the parent HTeO_4_^−^ anions by 193 nm laser irradiation. A similar two-photon photodetachment–photodissociation process has been observed in the PtI_3_^−^ anion [25]. To verify this hypothesis, we measured the photoelectron spectra of HTeO_4_^−^ under varying laser fluences, as shown in Figure 5. The relative intensity of the low-eBE band decreases significantly with reduced laser power, whereas the intensity of the X band remains largely unaffected. This trend confirms that the low-eBE band originates from a multiphoton process: the first photon induces photodissociation of the parent anion, while the second photon photodetaches an electron from the resulting fragment anion within the same ∼6 ns laser pulse duration. Resonance absorption calculations for HTeO_4_^−^ at 193 nm (Figure S4) support the feasibility of photodissociation and identify six possible photodissociation–photodetachment channels (Table S7). The EAs of O (1.461 eV [26]), H (0.754 eV [27]), and HO (1.828 eV [28]) do not match observed features and can be excluded. Among the remaining candidates, the dissociation channel producing TeO_3_^−^ has the lowest bond dissociation energy (2.838 eV), and its measured and calculated VDE closely matches the observed two-photo band. Therefore, we assign the two-photon photodissociation–photodetachment pathway as:

$${\mathrm{HTeO}}_{4}^{-}\;+\;h\nu \to {\mathrm{TeO}}_{3}^{-}\;+\;\mathrm{HO},$$

$${\mathrm{TeO}}_{3}^{-}\;+\;h\nu \;\to \;{\mathrm{TeO}}_{3}\;+\;e.$$

This assignment is further confirmed by bond order analysis (Figure S4), which shows that the Te–O(H) bond exhibits a significantly lower bond order (~65%) compared to the other three Te–O bonds, indicating it is the most susceptible to dissociation. In addition, very weak spectral signals, magnified by a factor of 30 and highlighted in the blue box in Figure 5, were observed in the 1.0–3.0 eV region of the HTeO_4_^−^ spectrum. However, due to their extremely low intensities, a definitive assignment remains elusive. Compared with the well-characterized photodetachment features of HSO_4_^−^, the photodissociation behavior of HTeO_4_^−^ highlights its inherent instability.

## 3. Materials and Methods

### 3.1. Experimental Methods

The experiments were conducted using a custom-built cryogenic anion cluster photoelectron spectroscopy (CRACPES) system consisting of an ESI source, a two-stage ion funnel, quadrupole mass spectroscopy, cryogenic ion traps, and a time-of-flight (TOF) mass spectrometer coupled to a magnetic-bottle negative-ion photoelectron spectrometer (NIPES), as previously described in detail [25,29]. The parent anion HTeO_4_^−^ was produced by electrospraying a 5 mM aqueous solution of H_6_TeO_6_ in a H_2_O:CH_3_CN = 1:2 mixture, and the TeO_2_^−^ and TeO_3_^−^ anions were generated via CID of HTeO_4_^−^ in the ion funnels by optimizing the corresponding radio frequency (RF) voltages and direct current (DC) gradients. The target anions were subsequently trapped and cooled to 13 K in a cryogenic 12-pole ion trap, followed by mass selection in the TOFMS. The anion pulses were decelerated before interacting with 193 nm (6.424 eV) laser pulses (ExciStar^TM^ XS 500, Coherent, Santa Clara, CA 95054, USA) in the photodetachment region. The photoelectrons were collected with near 100% efficiency using a magnetic-bottle photoelectron analyzer. The laser operated at a repetition rate of 50 Hz, and alternating laser-on/laser-off cycles were employed for shot-to-shot background subtraction. Raw photoelectron TOF data were converted to electron kinetic energy (eKE) spectra and calibrated using known spectra of I^−^ [30] and MnO_4_^−^ [31]. The final eBE spectra were obtained by subtracting the eKE spectra from the photon energy with an electron energy resolution (ΔE/E) of ∼1.9% (e.g., 30 meV @ 1.60 eV eKE).

### 3.2. Computational Details

All geometry optimizations were performed using DFT with the dispersion-corrected hybrid B3LYP-D3(BJ) [32,33,34,35,36,37] exchange correlation functional [32,33,34] and the aug-cc-pVTZ (for H and O atoms)/aug-cc-pVTZ-PP (for Te atom) basis sets [35,36,37], as implemented in Gaussian 16 software [38]. This functional was chosen for its good balance between computational cost and accuracy in modeling Te-containing systems [22,39]. Harmonic vibrational frequency analyses were performed to ensure that all optimized structures correspond to true minima (i.e., all frequencies are real). NPA [40] was performed for both anionic and neutral species at the same level of theory. The energies of the optimized low-lying anionic and neutral species were calculated using multiple methods, including IP-EOM-CCSD [41,42,43] and several DFT functionals: B3LYP-D3(BJ), M06-2X [44], PEB0-D3(BJ) [33,34,45], MN15L-D3(BJ) [33,34,46], ωB97XD [47], r2SCAN-3c [48,49], and TPSSh-D3(BJ) [33,34,50,51]. All calculations used the aug-cc-pVTZ(-PP) basis sets consistently. The DFT/r2SCAN-3c and IP-EOM-CCSD calculations were conducted using the ORCA 5.0 software [52], while the remaining calculations were performed with Gaussian 16. The VDEs were determined as the energy difference between the neutral and anionic species at the optimized geometry of the anion. The ADEs were defined as the energy differences between the optimized neutral and anion structures, including zero-point energy (ZPE) corrections. TDDFT calculations were used to compute vertical excitation energies with selected functionals (CAM-B3LYP [53], PEB0-D3(BJ), M06-2X, and ωB97XD), all employing the aug-cc-pVTZ(-PP) basis sets. Dyson orbitals were calculated at the IP-EOM-CCSD/aug-cc-pVTZ(-PP) level using the Q-Chem 5.0 program [54]. FCF simulations were performed with the ezSpectra program [55] using the optimized structures and vibration analysis obtained at the B3LYP-D3(BJ)/aug-cc-pVTZ(-PP) level. The absorption spectrum and bond dissociation energies were calculated at the B3LYP-D3(BJ)/aug-cc-pVTZ(-PP) level. The MOs, EDD, and bond order analyses were conducted with the Multiwfn code [56] and the corresponding isosurfaces were visualized using the VMD 1.9.3 program [57].

## 4. Conclusions

This study presents a combined experimental and theoretical investigation of the photoelectron spectroscopy of TeO_2_^−^, TeO_3_^−^, and HTeO_4_^−^ anions, which were generated via CID following ESI. The ADEs and VDEs determined from the experimental spectra agreed well with the theoretical values. Notably, TeO_2_^−^ and TeO_3_^−^ exhibited a significant multi-reference character. The ADEs of these anions were further refined using Franck–Condon factor (FCF) simulations. In particular, the significant structural change from TeO_3_^−^ (C_3v_) to TeO_3_ (D_3h_) upon photodetachment led to a large gap between its ADE and VDE, making experimental determination of the ADE challenging.

Analysis of photodetachment orbitals indicated that with the increasing oxygen content, the contribution of oxygen atoms to the detaching electrons in TeO_2_^−^, TeO_3_^−^, and HTeO_4_^−^ also increased. Additionally, a two-photon photodissociation–photodetachment channel was identified in the HTeO_4_^−^ anion, confirmed by photon-flux-dependent measurements. This work provides detailed insights into the electronic structures of these tellurium oxide anions, significantly enhancing our understanding of gas-phase chalcogen oxides and laying a solid experimental and theoretical foundation for the future synthesis and application of tellurium-based materials.

## Figures and Tables

**Table 1:** Experimental and calculated vertical detachment energies (VDEs) and adiabatic detachment energies (ADEs) for TeO_2_^−^, TeO_3_^−^, and HTeO_4_^−^. All theoretical calculations were performed with the aug-cc-pVTZ-(pp) basis set.

	VDE (eV)			ADE (eV)		
	TeO_2_^−^	TeO_3_^−^	HTeO_4_^−^	TeO_2_^−^	TeO_3_^−^	HTeO_4_^−^
Expt.	2.13	4.20	5.64	1.94	<4.01 ^a^	5.20
B3LYP-D3(BJ)	2.50	4.66	5.48	2.38	3.99	5.11
MN15L-D3(BJ)	2.23	4.29	5.34	2.06	3.52	5.00
r2SCAN-3c	2.14	4.32	4.96	2.09	3.68	4.77
ωB97XD	2.53	4.74	5.75	2.31	3.91	5.12
PEB0-D3(BJ)	2.53	4.68	5.34	2.33	3.87	5.00
M06-2X	2.77	4.96	6.14	2.50	3.97	5.27
TPSSh-D3(BJ)	2.34	4.43	5.17	2.23	3.75	4.88
IP-EOM-CCSD	2.36	4.48	5.76	/	/	/

^a^ The ADE of TeO_3_^−^ cannot be accurately determined experimentally due to the substantial geometric rearrangement upon the photodetachment.

**Table 2:** The charge distribution of TeO_2_^−/0^, TeO_3_^−/0^, and HTeO_4_^−/0^ based on NPA.

		Anion	Neutral	Δ
TeO_2_^−^	Te	0.00	0.76	0.76
	O	−0.50 × 2 ^a^	−0.38 × 2	0.24
TeO_3_^−^	Te	1.28	1.86	0.58
	O	−1.14 × 3 ^b^	−0.93 × 3	0.42
HTeO_4_^−^	H	0.47	0.49	0.02
	Te	2.99	2.94	−0.05
	O(1) ^c^	−1.04	−0.97	0.07
	O(2)	−1.16	−0.86	0.30
	O(3)	−1.13	−0.80	0.33
	O(4)	−1.13	−0.80	0.33

^a^ The charge on one of the 2 equivalent oxygen atoms multiplied by 2. ^b^ The charge on one of the 3 equivalent oxygen atoms multiplied by 3. ^c^ The atomic numbers are shown in Figure 2.

## Data Availability

All data included in this study are available upon request by contacting the corresponding author.

## Supplementary Materials

The following supporting information can be downloaded at: https://www.mdpi.com/article/10.3390/molecules30183757/s1. Figure S1: Mass spectrometry of TeO_2_^−^, TeO_3_^−^, and HTeO_4_^−^ anions. Figure S2: Molecular orbitals of TeO_2_^−^, TeO_3_^−^, and HTeO_4_^−^. Figure S3: Vibration modes of TeO_2_^−^, TeO_3_^−^, and HTeO_4_^−^ anions. Figure S4: The absorption spectrum and fuzzy bond order of HTeO_4_^−^. Table S1: Optimized coordinates of TeO_2_^−^, TeO_3_^−^, and HTeO_4_^−^ and corresponding neutrals. Table S2: T1 diagnostic values of TeO_2_^−^, TeO_3_^−^, and HTeO_4_^−^. Table S3: Vertical excitation energies of TeO_2_^−^, TeO_3_^−^, and HTeO_4_^−^. Table S4: Simulated FCF stick spectrum of TeO_2_^−^. Table S5: Simulated FCF stick spectrum of TeO_3_^−^. Table S6: Simulated FCF stick spectrum of HTeO_4_^−^. Table S7: Photodissociation–photodetachment channels of HTeO_4_^−^.

## Abbreviations

The following abbreviations are used in this manuscript:

ADE	adiabatic detachment energy
CID	collision-induced dissociation
CRACPES	cryogenic anion cluster photoelectron spectroscopy
DFT	density functional theory
EA	electron affinity
EDD	electron density difference
eBE	electron binding energy
eKE	electron kinetic energy
ESI	electrospray ionization
FCF	Frank–Condon factor
HOMO	highest occupied molecular orbital
MO	molecular orbital
NIPES	negative-ion photoelectron spectroscopy
NPA	natural population analysis
TDDFT	time-dependent density functional theory
TOF	time-of-flight
VDE	vertical detachment energy
RF	radio frequency
DC	direct current
