# Supplementary material for: Electronic Structures and Photodetachment of TeO2−, TeO3−, and HTeO4− Anions: A Cryogenic Photoelectron Spectroscopic Study
Source: Molecules. 2025 Sep 16;30(18):3757. doi: 10.3390/molecules30183757 (PMC12472665; doi:10.3390/molecules30183757)
Supplement: Supplementary file 1 [file molecules-30-03757-s001.zip › molecules-3839918-supplementary.pdf]

Supplementary Materials for

Electronic Structures and Photodetachment of TeO_2^-, TeO_3^-, and $HTeO_4^-$ Anions: A Cryogenic Photoelectron Spectroscopic Study

Fan Yang[1], Xueying Li[1], Peng Tang[1], Qixu Zhao[1], Jian Zhang[2], Ye Mei[1], Zhubin Hu[1], Zhenrong Sun[1,3,*], and Yan Yang[1,*]

[1]*State Key Laboratory of Precision Spectroscopy, School of Physics and Electronic Science, East China Normal University, Shanghai 200241, China.*

[2]*College of Chemistry & Chemical Engineering, Donghua University, Shanghai 201620, China.*

[3]*Collaborative Innovation Center of Extreme Optics, Shanxi University, Taiyuan, Shanxi 030006, China.*

List of Figures

Figure S1 Quadrupole mass spectrometry (a) and time-of-flight mass spectrometry (b) of TeO_2^-, TeO_3^-, and $HTeO_4^-$ anions. The blue sticks in (a) represent the corresponding isotopic simulation spectra. 2
Figure S2 Molecular orbitals of TeO_2^-, TeO_3^-, and $HTeO_4^-$ calculated at the B3LYP-D3(BJ)/aug-cc-pVTZ(-PP) level, with the molecular orbital components (%) labeled. 3
Figure S3 Atomic displacements of each vibrational mode for TeO_2^-, TeO_3^-, and $HTeO_4^-$ calculated at the B3LYP-D3(BJ)/aug-cc-pVTZ(-PP) level. 4
Figure S4 The absorption spectrum (a) and fuzzy bond order (b) of $HTeO_4^-$ calculated at the B3LYP-D3(BJ)/aug-cc-pVTZ(-PP) level. 5

List of Tables

Table S1 Optimized x,y,z coordinates in angstroms at B3LYP-D3(BJ)/aug-cc-pVTZ(-PP) level for TeO_2^-, TeO_3^- , $HTeO_4^-$, and corresponding neutrals. 6
Table S2 T1 diagnostic values of TeO_2^-, TeO_3^- , $HTeO_4^-$ anions. 7
Table S3 Experimentally measured eBEs and relative eBEs and theoretically calculated excitation energies of neutral excited states from TDDFT calculations. 7
Table S4 Simulated FCF stick spectrum for TeO_2^- (doublet ground state) to TeO_2 (singlet ground state). 8
Table S5 Simulated FCF stick spectrum for TeO_3^- (doublet ground state) to TeO_3 (singlet ground state). 9
Table S6 Simulated FCF stick spectrum for $HTeO_4^-$ (singlet ground state) to $HTeO_4$ (doublet ground state). 10
Table S7 Photodissociation–photodetachment channels of $HTeO_4^-$, with calculated dissociation energies at the B3LYP-D3(BJ)/aug-cc-pVTZ(-PP) level and experimental VDE/ADE of product anions. 11

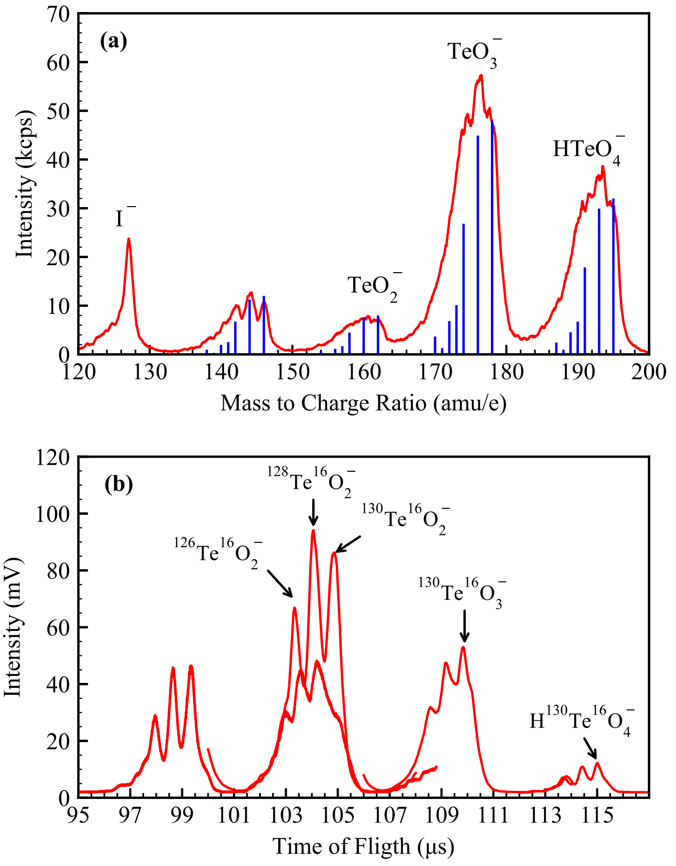

Figure S1: Quadrupole mass spectrometry (a) and time-of-flight mass spectrometry (b) of TeO_2^-, TeO_3^-, and $HTeO_4^-$ anions. The blue sticks in (a) represent the corresponding isotopic simulation spectra.

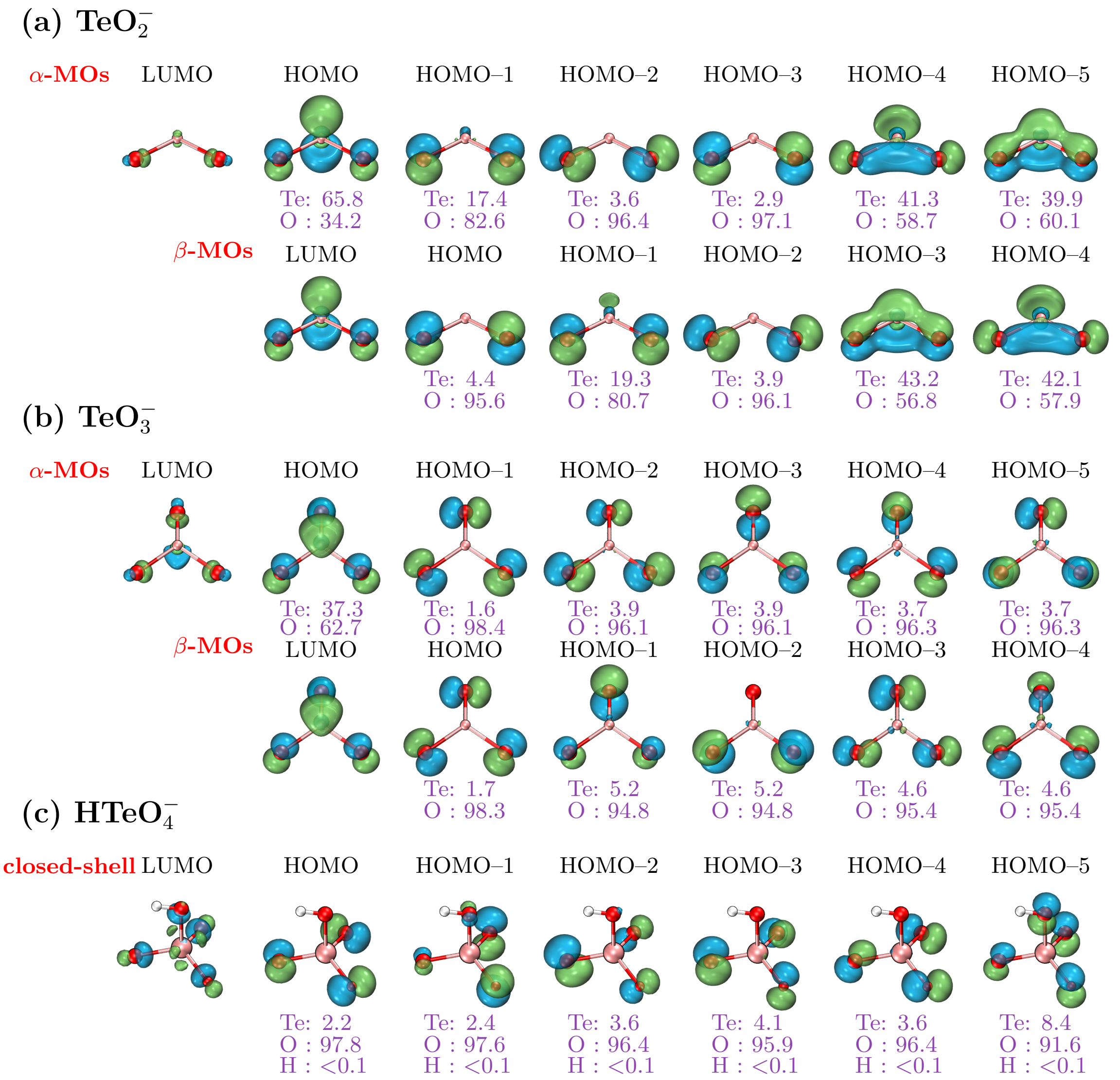

Figure S2: Molecular orbitals of TeO_2^-, TeO_3^-, and $HTeO_4^-$ calculated at the B3LYP-D3(BJ)/aug-cc-pVTZ(-PP) level, with the molecular orbital components (%) labeled.

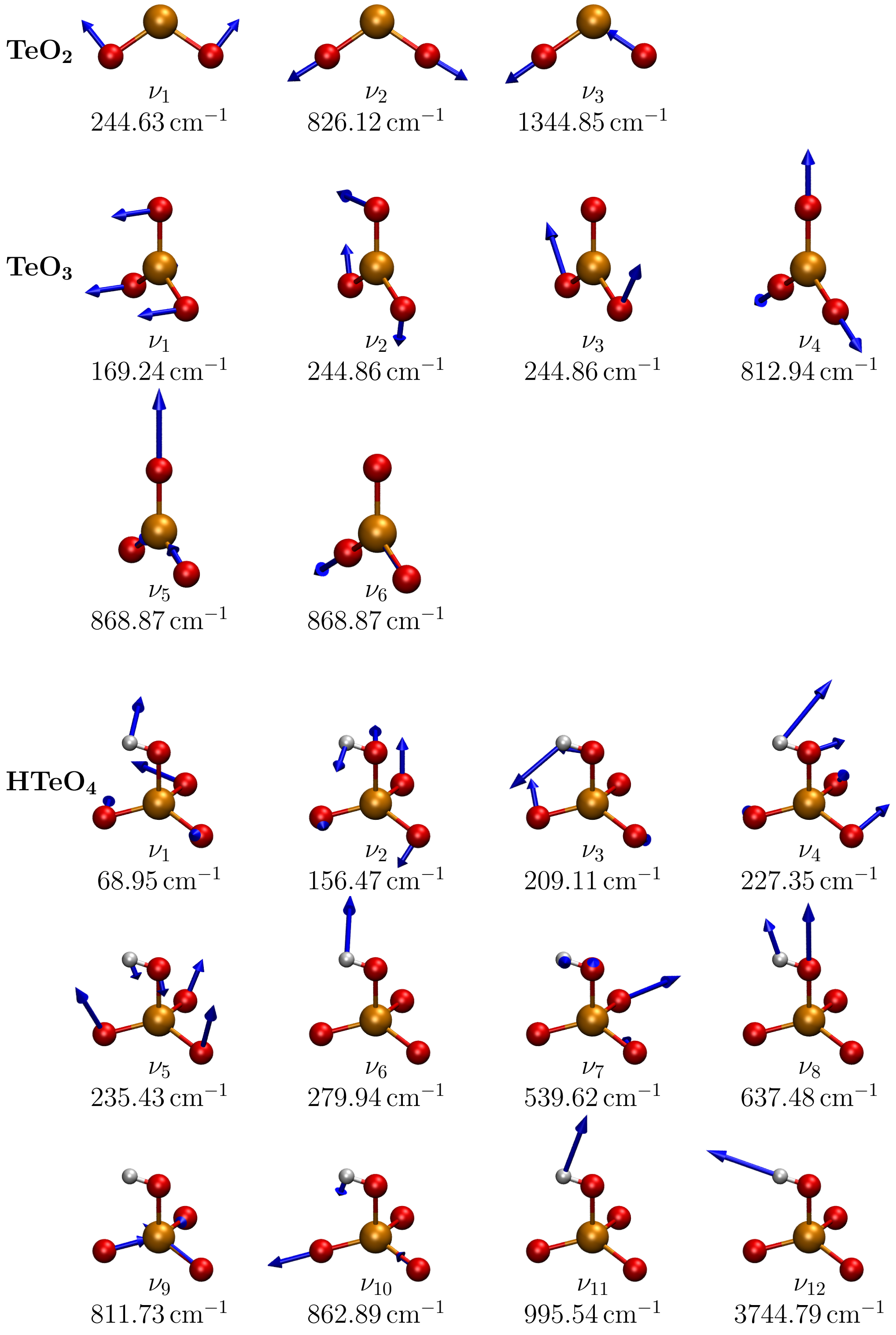

Figure S3: Atomic displacements of each vibrational mode for TeO_2^-, TeO_3^-, and $HTeO_4^-$ calculated at the B3LYP-D3(BJ)/aug-cc-pVTZ(-PP) level.

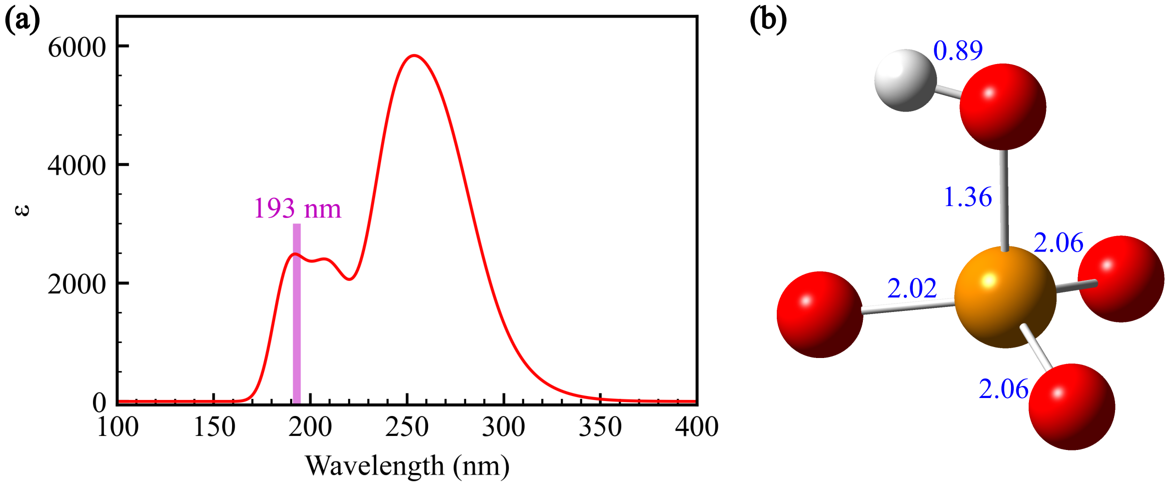

Figure S4: The absorption spectrum (a) and fuzzy bond order (b) of $HTeO_4^-$ calculated at the B3LYP-D3(BJ)/aug-cc-pVTZ(-PP) level.

Table S1: Optimized x,y,z coordinates in angstroms at B3LYP-D3(BJ)/aug-cc-pVTZ(-PP) level for TeO_2^-, TeO_3^-, $HTeO_4^-$, and corresponding neutrals.

TeO_2^-	Te	0.00000000	0.00000000	0.25212400
	O	0.00000000	1.52754000	-0.81940200
	O	0.00000000	-1.52754000	-0.81940200
TeO_2	Te	0.00000000	0.00000000	0.24121700
	O	0.00000000	1.48481000	-0.78395600
	O	0.00000000	-1.48481000	-0.78395600
TeO_3	Te	0.00000000	0.00000000	0.17791900
	O	0.00000000	1.76370300	-0.38549100
	O	-1.52741200	-0.88185200	-0.38549100
	O	1.52741200	-0.88185200	-0.38549100
TeO_3	Te	0.00000000	0.00000000	0.00011800
	O	0.00000000	1.79819600	-0.00025500
	O	-1.55728300	-0.89909800	-0.00025500
	O	1.55728300	-0.89909800	-0.00025500
$HTeO_4^-$	H	-2.14846900	0.00507600	-0.82325600
	Te	0.08593800	-0.00013900	0.06287700
	O	1.01098300	1.53144000	-0.25389500
	O	1.00582500	-1.53360700	-0.26159600
	O	-1.32896100	0.00539900	-1.33488100
	O	-0.97788400	-0.00296200	1.54457600
$HTeO_4$	H	1.85093800	1.46079100	0.21605900
	Te	-0.00598800	-0.07357100	-0.06588800
	O	-1.42364900	0.22080300	-1.13511300
	O	-0.94247400	-0.22279700	1.60986200
	O	0.89602300	1.61600100	0.15044500
	O	1.27765400	-1.31839400	-0.22393000

Table S2: T1 diagnostic values of TeO_2^-, TeO_3^- , $HTeO_4^-$ anions.

	TeO_2^-	TeO_3^-	$HTeO_4^-$
CCSD (T1Diag)	0.026	0.028	0.020
IP-EOM-CCSD	0.025	0.028	0.020

Basis set: aug-cc-pVTZ(-PP).

Table S3: Experimentally measured eBEs and relative eBEs and theoretically calculated excitation energies of neutral excited states from TDDFT calculations.

		Expt. (eV)		Calc. (eV) [a]			
	Peaks	eBE	ΔeBE	CAM-B3lYP-D3(BJ)	M06-2X	PBE0-D3(BJ)	ωB97X-D
TeO_2^-	X	2.13	0	0	0	0	0
	A	4.04	1.91	2.18	2.07	2.16	2.19
	B	4.28	2.22	2.23	2.19	2.18	2.24
	C	5.91	3.78	3.47	3.44	3.52	3.52
TeO_3^-	X	4.20	0	0	0	0	0
	A	5.25	1.05	1.05	1.05	0.93	1.03
	B	5.46	1.26	1.36	1.26	1.28	1.36
				1.36	1.26	1.28	1.36
$HTeO_4^-$	X	5.64	0	0	0	0	0
				0.13	0.26	0.23	0.13
				0.34	/	0.33	0.28

[a] Basis set: aug-cc-pVTZ(-PP).

Table S4: Simulated FCF stick spectrum for TeO_2^- (doublet ground state) to TeO_2 (singlet ground state).

eBE(eV)	Intensity	FCF	Vibronic Transition
2.032	2.64E-01	5.14E-01	0(0)->1(0)
2.135	3.10E-01	5.57E-01	0(0)->1(1v2)
2.237	2.05E-01	4.53E-01	0(0)->1(2v2)
2.340	9.96E-02	3.16E-01	0(0)->1(3v2)
2.442	3.95E-02	1.99E-01	0(0)->1(4v2)
2.544	1.35E-02	1.16E-01	0(0)->1(5v2)
2.647	4.13E-03	6.42E-02	0(0)->1(6v2)
2.749	1.15E-03	3.39E-02	0(0)->1(7v2)
2.063	6.38E-03	-7.99E-02	0(0)->1(1v1)
2.366	1.17E-02	1.08E-01	0(0)->1(2v3)
2.165	4.71E-03	-6.86E-02	0(0)->1(1v1,1v2)
2.195	1.15E-03	-3.39E-02	0(0)->1(2v1,1v2)
2.267	1.83E-03	-4.28E-02	0(0)->1(1v1,2v2)
2.468	1.37E-02	1.17E-01	0(0)->1(1v2,2v3)
2.571	9.07E-03	9.52E-02	0(0)->1(2v2,2v3)
2.673	4.41E-03	6.64E-02	0(0)->1(3v2,2v3)
2.775	1.75E-03	4.18E-02	0(0)->1(4v2,2v3)

Table S5: Simulated FCF stick spectrum for TeO_3^- (doublet ground state) to TeO_3 (singlet ground state).

eBE(eV)	Intensity	FCF	Vibronic Transition	eBE(eV)	Intensity	FCF	Vibronic Transition
3.951	0.00147	0.0383	0(0)->1(16v1)	4.257	0.00114	-0.0338	0(0)->1(21v1,2v4)
3.972	0.00297	-0.0545	0(0)->1(17v1)	4.278	0.00198	0.0445	0(0)->1(22v1,2v4)
3.993	0.00555	0.0745	0(0)->1(18v1)	4.299	0.00321	-0.0567	0(0)->1(23v1,2v4)
4.014	0.00966	-0.0983	0(0)->1(19v1)	4.320	0.00489	0.0699	0(0)->1(24v1,2v4)
4.035	0.01565	0.1251	0(0)->1(20v1)	4.341	0.00697	-0.0835	0(0)->1(25v1,2v4)
4.056	0.02367	-0.1538	0(0)->1(21v1)	4.362	0.00932	0.0965	0(0)->1(26v1,2v4)
4.077	0.03344	0.1829	0(0)->1(22v1)	4.383	0.01169	-0.1081	0(0)->1(27v1,2v4)
4.098	0.04422	-0.2103	0(0)->1(23v1)	4.404	0.01376	0.1173	0(0)->1(28v1,2v4)
4.119	0.05478	0.2341	0(0)->1(24v1)	4.425	0.01519	-0.1233	0(0)->1(29v1,2v4)
4.140	0.06362	-0.2522	0(0)->1(25v1)	4.446	0.01574	0.1254	0(0)->1(30v1,2v4)
4.161	0.06932	0.2633	0(0)->1(26v1)	4.467	0.01529	-0.1236	0(0)->1(31v1,2v4)
4.182	0.07089	-0.2662	0(0)->1(27v1)	4.488	0.01392	0.1180	0(0)->1(32v1,2v4)
4.203	0.06804	0.2608	0(0)->1(28v1)	4.509	0.01188	-0.1090	0(0)->1(33v1,2v4)
4.224	0.06130	-0.2476	0(0)->1(29v1)	4.530	0.00949	0.0974	0(0)->1(34v1,2v4)
4.245	0.05182	0.2276	0(0)->1(30v1)	4.551	0.00708	-0.0842	0(0)->1(35v1,2v4)
4.266	0.04107	-0.2027	0(0)->1(31v1)	4.572	0.00494	0.0703	0(0)->1(36v1,2v4)
4.287	0.03050	0.1747	0(0)->1(32v1)	4.593	0.00320	-0.0566	0(0)->1(37v1,2v4)
4.308	0.02120	-0.1456	0(0)->1(33v1)	4.614	0.00193	0.0439	0(0)->1(38v1,2v4)
4.329	0.01376	0.1173	0(0)->1(34v1)	4.421	0.00118	-0.0343	0(0)->1(24v1,3v4)
4.350	0.00833	-0.0913	0(0)->1(35v1)	4.442	0.00179	0.0423	0(0)->1(25v1,3v4)
4.371	0.00469	0.0685	0(0)->1(36v1)	4.463	0.00254	-0.0503	0(0)->1(26v1,3v4)
4.392	0.00244	-0.0494	0(0)->1(37v1)	4.484	0.00338	0.0581	0(0)->1(27v1,3v4)
4.413	0.00117	0.0343	0(0)->1(38v1)	4.505	0.00423	-0.0650	0(0)->1(28v1,3v4)
4.115	0.00135	0.0367	0(0)->1(19v1,1v4)	4.526	0.00497	0.0705	0(0)->1(29v1,3v4)
4.136	0.00262	-0.0512	0(0)->1(20v1,1v4)	4.547	0.00549	-0.0741	0(0)->1(30v1,3v4)
4.157	0.00472	0.0687	0(0)->1(21v1,1v4)	4.568	0.00570	0.0755	0(0)->1(31v1,3v4)
4.178	0.00785	-0.0886	0(0)->1(22v1,1v4)	4.589	0.00556	-0.0746	0(0)->1(32v1,3v4)
4.199	0.01212	0.1101	0(0)->1(23v1,1v4)	4.610	0.00509	0.0714	0(0)->1(33v1,3v4)
4.220	0.01740	-0.1319	0(0)->1(24v1,1v4)	4.631	0.00437	-0.0661	0(0)->1(34v1,3v4)
4.241	0.02327	0.1526	0(0)->1(25v1,1v4)	4.652	0.00352	0.0593	0(0)->1(35v1,3v4)
4.262	0.02905	-0.1705	0(0)->1(26v1,1v4)	4.673	0.00265	-0.0515	0(0)->1(36v1,3v4)
4.283	0.03389	0.1841	0(0)->1(27v1,1v4)	4.694	0.00186	0.0432	0(0)->1(37v1,3v4)
4.304	0.03695	-0.1922	0(0)->1(28v1,1v4)	4.606	0.00115	0.0339	0(0)->1(28v1,4v4)
4.325	0.03770	0.1942	0(0)->1(29v1,1v4)	4.627	0.00143	-0.0378	0(0)->1(29v1,4v4)
4.346	0.03600	-0.1897	0(0)->1(30v1,1v4)	4.648	0.00167	0.0408	0(0)->1(30v1,4v4)
4.367	0.03216	0.1793	0(0)->1(31v1,1v4)	4.669	0.00183	-0.0428	0(0)->1(31v1,4v4)
4.387	0.02688	-0.1640	0(0)->1(32v1,1v4)	4.690	0.00189	0.0435	0(0)->1(32v1,4v4)
4.408	0.02101	0.1449	0(0)->1(33v1,1v4)	4.711	0.00183	-0.0428	0(0)->1(33v1,4v4)
4.429	0.01533	-0.1238	0(0)->1(34v1,1v4)	4.732	0.00167	0.0409	0(0)->1(34v1,4v4)
4.450	0.01044	0.1022	0(0)->1(35v1,1v4)	4.753	0.00143	-0.0379	0(0)->1(35v1,4v4)
4.471	0.00661	-0.0813	0(0)->1(36v1,1v4)	4.774	0.00115	0.0340	0(0)->1(36v1,4v4)
4.492	0.00389	0.0624	0(0)->1(37v1,1v4)				
4.513	0.00212	-0.0460	0(0)->1(38v1,1v4)				
4.534	0.00106	0.0326	0(0)->1(39v1,1v4)				

Table S6: Simulated FCF stick spectrum for $HTeO_4^-$ (singlet ground state) to $HTeO_4$ (doublet ground state).

$j\nu_7$, j = / $i\nu 4$, i =	1	2	3	4	5	6	7	8	9	10
1										
2			2.20E-06	3.59E-06	4.43E-06	4.29E-06	3.32E-06	2.09E-06	1.07E-06	
3		3.04E-06	6.83E-06	1.09E-05	1.31E-05	1.24E-05	9.31E-06	5.67E-06	2.81E-06	1.12E-06
4	2.03E-06	7.01E-06	1.54E-05	2.40E-05	2.82E-05	2.58E-05	1.89E-05	1.11E-05	5.29E-06	2.03E-06
5	3.70E-06	1.25E-05	2.69E-05	4.09E-05	4.68E-05	4.16E-05	2.95E-05	1.67E-05	7.64E-06	2.79E-06
6	5.45E-06	1.81E-05	3.78E-05	5.60E-05	6.22E-05	5.37E-05	3.67E-05	2.01E-05	8.77E-06	3.04E-06
7	6.65E-06	2.15E-05	4.39E-05	6.33E-05	6.83E-05	5.70E-05	3.76E-05	1.97E-05	8.19E-06	
8	6.84E-06	2.16E-05	4.29E-05	6.01E-05	6.28E-05	5.06E-05	3.21E-05	1.61E-05		
9	6.03E-06	1.85E-05	3.58E-05	4.86E-05	4.91E-05	3.81E-05	2.31E-05			
10	4.59E-06	1.37E-05	2.57E-05	3.37E-05	3.28E-05	2.45E-05				
11	3.04E-06	8.82E-06	1.60E-05	2.03E-05	1.90E-05					
12	1.76E-06	4.95E-06	8.66E-06	1.06E-05						
13		2.43E-06	4.09E-06							
14		1.05E-06								

[a] The intensity of the 0(0)->1(0) vibronic transition is 6.649e-10, and other FCFs with weaker intensity are ignored.
[b] The eBE of the 0(0)->1(0) vibronic transition (equivalent to EA) is 5.1092 eV.
[c] The eBE of the 0(0)->($i\nu_3$, $j\nu_6$) vibronic transition: EA + 0.028188*i + 0.066905*j eV.
[a] The vibration modes start from 1, as shown in Figure S3.

Table S7: Photodissociation–photodetachment channels of $HTeO_4^-$, with calculated dissociation energies at the B3LYP-D3(BJ)/aug-cc-pVTZ(-PP) level and experimental VDE/ADE of product anions.

Channel No.	Photodissociation	Dissociation Energy (eV)	Photodetachment	Experimental VDE/ADE (eV)
1	$HTeO_4^- + h\nu \rightarrow TeO_3^- + HO$	2.838	$TeO_3^- + h\nu \rightarrow TeO_3 + e^-$	4.20 [a]
2	$HTeO_4^- + h\nu \rightarrow TeO_3 + HO^-$	/	$OH^- + h\nu \rightarrow HO + e^-$	1.828 [b]
3	$HTeO_4^- \rightarrow TeO_4^- + H$	4.753	$TeO_4^- + h\nu \rightarrow H + e^-$	/
4	$HTeO_4^- \rightarrow TeO_4 + H^-$	/	$H^- + h\nu \rightarrow H + e^-$	0.754 [c]
5	$HTeO_4^- \rightarrow HTeO_3^- + O$	5.813	$HTeO_3^- + h\nu \rightarrow HTeO_3 + e^-$	/
6	$HTeO_4^- \rightarrow HTeO_3 + O^-$	/	$O^- + h\nu \rightarrow O + e^-$	1.461 [d]

[a] Experimental VDE of TeO_3^-
[b] Experimental EA of OH from Ref [3]
[c] Experimental EA of H from Ref [2]
[d] Experimental EA of O from Ref [1]

References

[1] W. Chaibi, R. J. Peláez, C. Blondel, C. Drag, and C. Delsart. Effect of a magnetic field in photodetachment microscopy. *The European Physical Journal D*, 58(1):29–37, April 2010.

[2] C.R. Quick, J.B. Donahue, Stanley Cohen, H.C. Bryant, C.Y. Tang, P.G. Harris, A.H. Mohagheghi, R.A. Reeder, H. Sharifian, H. Toutounchi, and W.W. Smith. Photodetachment of the H ion. *Nuclear Instruments and Methods in Physics Research Section B: Beam Interactions with Materials and Atoms*, 5657:205–210, May 1991.

[3] P. A. Schulz, Roy D. Mead, P. L. Jones, and W. C. Lineberger. OH^- and OD threshold photodetachment. *The Journal of Chemical Physics*, 77(3):1153–1165, August 1982.
